# Improvement on Thermostability of Pectate Lyase and Its Potential Application to Ramie Degumming

**DOI:** 10.3390/polym14142878

**Published:** 2022-07-15

**Authors:** Huan Xu, Xiangyuan Feng, Qi Yang, Ke Zheng, Le Yi, Shengwen Duan, Lifeng Cheng

**Affiliations:** Institute of Bast Fiber Crops, Chinese Academy of Agricultural Science, Changsha 410205, China; xuhuan0310@yeah.net (H.X.); fengxiangyuan@caas.cn (X.F.); yangqi@caas.cn (Q.Y.); zhengke@caas.cn (K.Z.); yile0219@yeah.net (L.Y.)

**Keywords:** *Dickeya dadantii* DCE-01, pectate lyase, ramie, degumming, site-directed mutation, thermostability

## Abstract

In order to obtain a thermostable pectate lyase for ramie degumming, a rational design based on structural analysis was carried out on a novel pectate lyase (Pel419) derived from the *Dickeya Dadantii* DCE-01 for high-efficiency ramie degumming. A total of five potential amino acid sites were chosen to replace residues. Then, the mutant enzymes were subjected to the heterologous expressions in *Escherichia coli* and their enzymatic characteristics were determined. The optimal reaction temperature for the five mutants kept consistent with that for the wild type. The enzyme activity and thermal stability of mutant V52A were significantly improved. Meanwhile, the weight loss rate obtained by V52A with the best enzymatic characteristics in the ramie degumming process at 50 °C is comparable with that obtained by commercial cotton-ramie processing pectinases, indicating that V52A was a potential industrial enzyme that could be applied to large-scale ramie degumming. In this study, the biological functions of conservative residues of Pel419 were preliminarily explored. The mutant V52A with both enzymatic activity and improved heat resistance was acquired, providing a superior material for developing enzyme preparations of ramie degumming, and rendering an effective method for the rational design aiming to improve the thermostability of pectate lyase.

## 1. Introduction

As an important constituent member of the pectinase enzyme family, pectate lyase (Pel, EC 4.2.2.2) can randomly cut the main pectin chain via a trans-elimination effect and then generate oligo-galacturonic acids [1]. It has been widely explored and used in papermaking, textile industries and the food industry since it can serve as a substitutive countermeasure for chemical processing to relieve environmental pollution [2]. By virtue of high activity in an alkaline environment, pectate lyase has been extensively applied to the degradation of pectic substances in various fields such as textiles, food, papermaking and environmental protection, and it is more common in bast fiber degumming and cotton-ramie refining in the textile industry [3,4,5,6,7]. If applied to ramie bio-degumming, alkaline pectate lyase will yield high efficiency, low cost and low pollution. Moreover, it can reserve the inherent morphology of ramie fibers and improve their quality [8]. In addition, research findings show that Ca^2+^ can bind to pectate lyase and substrates to promote the enzyme-substrate reaction, so as to improve the activity of pectate lyase [9,10].

The high cost of industrial degumming enzymes and their low performance under extreme conditions have been considered as the main obstacles impeding their industrial application [2,11]. Given the latest development of pectate lyase, the industrial production can be significantly promoted by improving the catalytic efficiency and high-temperature resistance of enzymes. The ramie degumming high-efficiency strain *Dickeya dadantii* DCE-01 produces multiple pectate lyases, such as Pel419, Pel4J4 and PelG403, among which Pel419 has superior activity and favorable stability, but its thermostability remains to be further improved for the sake of bio-degumming [12].

With the development of protein engineering techniques, the protein rational design, an effective genetic method optimizing protein characteristics, is conducive to generating mutations with strengthened characteristics, and meanwhile, expounding the structure-function relationship of enzymes [13,14,15]. Recently, more attention has been paid to conservative catalytic residues that play important roles in regulating enzymatic structure and catalytic performance [16]. For example, Chen et al. used homologous modeling to rationally design glycosyltransferase. A mutant with high thermal stability was obtained, and its optimum catalytic temperature was increased from 35 °C to 40 °C [17]. However, the noncatalytic potential residues non-conservative for proteins have been reported in few studies. In this study, efforts were made to improve the rigidity of the whole protein structure and enhance the thermostability of the enzyme by strengthening the stability of secondary structure in the noncatalytic protein region.

In a previous study, *pel*419 (GenBank ID: JX964997) was identified from the ramie degumming high-efficiency strain *D. dadantii* DCE-01 and cloned onto pET28a, which was a member of the Pec lyase C family with favorable pectate lyases activity and thermostability [12]. In this study, site-directed mutation was performed for the noncatalytic residues in the nonconservative region of Pel419, expecting to acquire good mutants with improved thermostability, obtain ideal materials for developing ramie degumming enzyme preparations and provide a promising candidate method for the large-scale biotechnology applications aiming to improve the thermostability of pectate lyase.

## 2. Materials and Methods

### 2.1. Materials

The recombinant plasmid pET28a-*pel*419 was constructed and preserved by Functional Fiber Material Laboratory, Institute of Bast Fiber Crops, Chinese Academy of Agricultural Sciences. *Escherichia coli* DH5α (TsingKe, Beijing, China) was used for gene cloning and *E. coli* BL21(DE3) (TsingKe, Beijing, China) for the expressions of recombinant proteins. The rapid site-directed mutation kit (Tiangen, Beijing, China) was used to introduce single mutation sites.

A protein quantification kit, Kan mycin, isopropyl-β-d-thiogalactoside (IPTG) and restriction endonuclease were purchased from Tiangen (Beijing, China). Primer synthesis & purification and DNA sequencing were completed by TsingKe (Changsha, China). Raw ramie (China ramie No.1) was gifted by the Perennial Breeding Laboratory, Institute of Bast Fiber Crops, Chinese Academy of Agricultural Sciences.

### 2.2. Methods

#### 2.2.1. Prediction of Mutation Sites

Pectate lyases from bacterial were selected in the PDB database as the template. Next, homology modeling was implemented using SWISS-MODEL (https://swissmodel.expasy.org/ accessed on 8 February 2022), and the potential unstable amino acid sites with high temperature B-factor in the protein structure were predicted via B-FITTER (Table S1) [18]. Conservative regions such as active sites and substrate binding pockets of the Pel419 structure were determined through multiple sequence alignment and Pymol visualization analysis. It was generally thought that the amino acids at these sites and regions were correlated with the enzymatic catalytic function, so these sites were avoided, whereas the secondary structural sites in the noncatalytic region were chosen for the mutation. Afterwards, the mutation sites in the Pel419 structure were substituted by the biological software SPDBV(Version 4.0, ExPASy, Geneva, Switzerland), followed by the energy minimization, thus determining the mutant structure.

#### 2.2.2. Construction and Prokaryotic Expression of Recombinant Mutagenic Plasmid, Preparation of Cell-Free Extract

The recombinant plasmid pET28a-*pel*419 was taken as the template, and the whole-plasmid PCR was performed using the rapid site-directed mutation kit and the primers in Table S2 to introduce the target amino acid site. Next, the enzyme digestion of the PCR product was conducted through the restriction endonuclease *Dpn* I to remove the plasmid template, and the obtained PCR product was transformed into *E. coli* DH5α. Afterwards, positive transformants were screened on the LB agar plate containing 100 μg/mL kanamycin for the sake of plasmid extraction and DNA sequencing. The verified recombinant plasmid was transformed into *E. coli* BL21(DE3), then mutant engineering bacteria were taken and inoculated into 200 mL LB culture medium (50 μg/mL Kan) until *OD*_600_ reached about 0.4–0.6, and then IPTG was added with the final concentration of 0.5 mmol/L for enzyme induction under the optimal shake-flask culture conditions(28 °C,120 r/min).

#### 2.2.3. SDS-PAGE Analysis and Enzymatic Activity Determination

A part of fermentation broth was taken and bacterial cells were collected. Another part of fermentation broth was operated using an ultrasonic cell disruption system at 300 W for 30 s with an interval of 20 s, lasting 20 min in total, followed by the centrifugation at 9000 *g* for 5 min and the supernatant (periplasmic enzyme) was collected and properly diluted using a buffer solution for the enzymatic activity determination.

SDS-PAGE was performed for the completely induced bacterial cells by reference to the Laemm1i method [19]. The fermentation broth was treated with the ultrasonic cell disruption system, then the supernatant was collected, in which the protein content was determined via the Bradford method, and the activity of pectate lyase was measured as the release of unsaturated oligogalacturonates during cleavage of polygalacturonic acid (PGA) [20,21]. The Gly-NaOH buffer solution (pH 9.0, 0.05 mol/L) was used to prepare polygalacturonic acid sodium solution (5 mg/mL) and CaCl_2_ was added until the final concentration of 1 mmol/L. Enzyme solutions were added to 1 mL substrate solution and incubated at 50 °C for 10 min. After the mixture was cooled to an ambient temperature, absorbance of the mixture at 235 nm was determined after the reaction was terminated by adding 3 mL of 0.03 mol/L H_3_PO_4_. The experiment was carried out using inactive enzymes in the control group. The unit (U) of enzymes was defined as the enzymatic quantity when 1 μmol of unsaturated revertose was released per min under the determination conditions [22]. All experiments were implemented in triplicate.

#### 2.2.4. Thermostability of Mutants

The enzymatic activity of Pel419 mutants in the enzyme solution was determined at 35, 40, 45, 50, 55, 60 and 65 °C to determine the optimal enzymatic hydrolysis temperature. Then, the temperature was held at 50 °C for 1 h, and the thermostability was observed by determining the residual enzymatic activity and evaluated according to the ratio of residual activity to the initial activity. In addition, the half-life period (t_1/2_) was determined at 50 °C, which was defined as the time when the enzymatic activity declined to the half of the initial activity at a specific temperature.

#### 2.2.5. Ramie Degumming through Enzymatic Method

In total, 20 g of shell-less ramie was accurately weighed and placed into a 500 mL shake flask. Next, the wild enzyme and V52A were taken for ramie degumming. The pectate lyase (Novozymes, BioPrep) produced for commercial ramie degumming was taken as the positive control and treated ramie with distilled water as a negative control (CK). Afterwards, each enzyme solution was diluted using the buffer solution (pH 9.0) until the final enzymatic activity of 100 U/mL. The CaCl_2_ was added until reaching the final concentration of 1 mmol/L, and then the temperature was regulated to 50 °C. The diluted pectate lyase solution was taken for ramie soaking at the proportion of 10:1. After the oscillation reaction at 50 °C for 2 h, a temperature of 105 °C was held for 20 min, and thus ramie degumming was terminated.

Morphological observation of fibers: The ramie fibers of raw ramie and those after degumming through the enzymatic method were taken, followed by metal spraying. The longitudinal surface morphologies of the fibers were observed via scanning electron microscope (SEM, Hitachi S-4800)

Determination of weight loss rate [23]: The mass of the raw ramie and fermented ramie were set as *G_m_* and *G_f_*, respectively, and the weight loss rate was calculated through the following formula:

$$\mathrm{Weight}\;\mathrm{loss}\;\mathrm{rate}\;\%=\frac{G_{m}-G_{f}}{G_{m}}\times 100$$

## 3. Results

### 3.1. Selection of Mutation Sites

**Figure 1:**
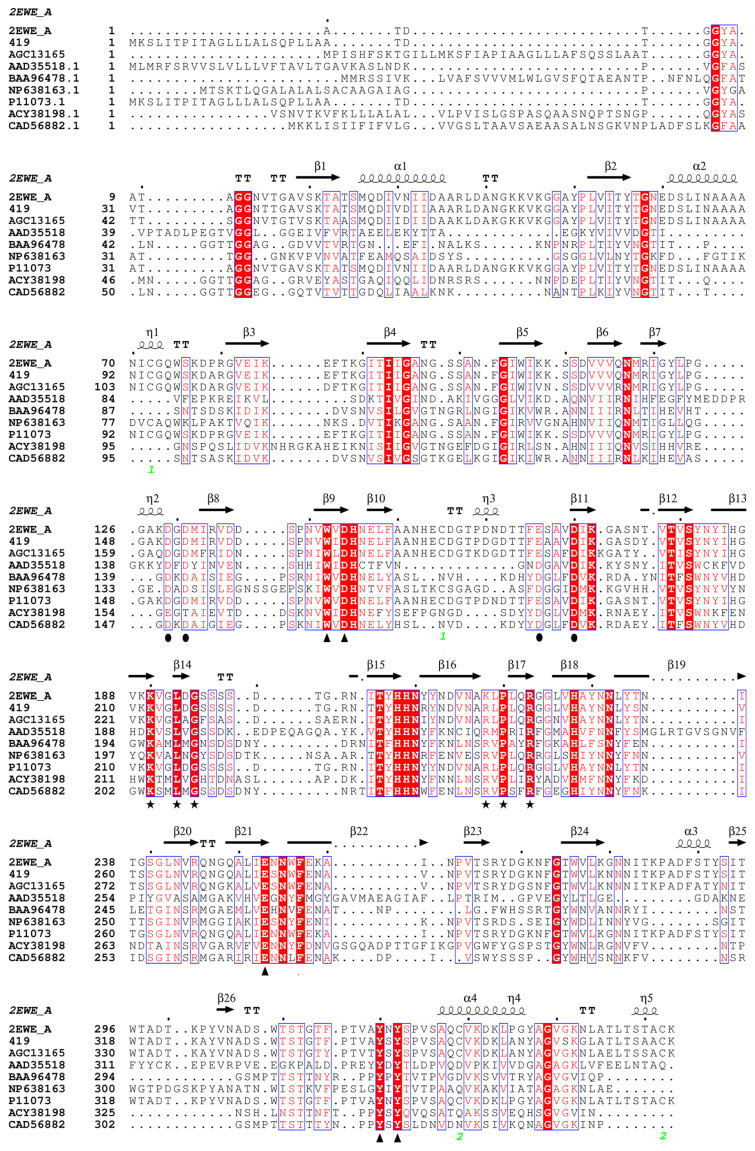
Sequence alignment of pectate lyases from different microbial sources. The circle represents the Ca^2+^ binding site, the five-pointed star and its vicinity represent the site related to the substrate binding pocket, and the triangle represents the important conserved amino acid site. Two sets of green numbers represent two pairs of disulfide bonds. Namely 2EWE_A (PDB ID), AGC13165 (GenBank), AAD35518 (GenBank), BAA96478 (GenBank), NP638163 (GenBank), P11073 (UniProt), ACY38198 (GenBank) and CAD56882 (GenBank).

**Figure 2:**
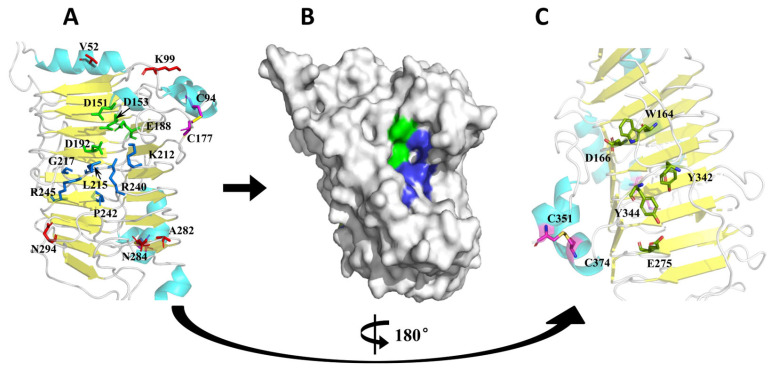
Pel419 conserved site. (**A**) Ca^2+^ binding site; the green and dark blue sticks indicate calcium-binding and catalytic residues in the active center, respectively. The red bars represent the selected mutation sites. (**B**) Substrate binding pocket; green and dark blue indicate the calcium ion-binding site and catalytic site of the active center, respectively. (**C**) Highly conserved site; dark green bars correlate with catalytic activity. All of the structural diagrams were drawn using PyMOL software (Version 2.4, Schrödinger, New York, NY, USA).

The Pel from *Dickeya chrysanthemi* (PDB: 2EWE) with the amino acid sequence homology as high as 89% was taken as the template for homology modeling, and the spatial structure of Pel419 was acquired [24]. By combining the amino acid sequence alignment and spatial structure analysis [2] (Figure 1 and Figure 2), D151, D153, E188, D192 and their nearby regions were obtained as the Ca^2+^ binding sites of Pel419, which formed substrate binding pockets with G200, K212, C215, R240, P242 and R245. All these binding pockets were exposed on the surface of the 3D space (Figure 2A,B). Meanwhile, it was speculated that the highly conservative W164, D166, E275, Y342 and Y344 were closely related to the catalytic activity of enzymes (Figure 2C). Moreover, the stability of Pel419 was positively influenced by the two pairs of disulfide bonds formed between C94 and C177 and between C351 and C374 (Figure 2A,C). All of these sites were important potential sites influencing the catalytic function of Pel419, so they should be avoided in the rational design aiming to improve the thermostability. By combining the potential unstable amino acid sites with high B-factors in the 3D structure Pel419, the conservative regions and disulfide bonds were excluded, a total of 5 non-catalytic mutation sites, V52, K99, A282, N284 and N294, were chosen to detect the influence of mutation on the thermostability of enzymes and three strategies were adopted. Firstly, low molecular weight amino acids in the flexile region were replaced by high molecular weight ones to enhance their rigidity. Alanine mutated into valine (A282V) and lysine mutated into arginine (K99R). Secondly, the amino acids, to the disadvantage of the α-helix stability in the rigid region, were replaced by those contributing to the stronger α-helix stability. The valine of α-helix mutated into alanine (V52A). Thirdly, charged amino acids were introduced to enhance the effect of ionic bonds and strengthen their stability. Asparagine was mutated into aspartic acids (N284D and N294D). The recombinant plasmid pET28a-pel419 was taken as the template for whole-plasmid PCR to introduce the target amino acid site (Figure S2).

### 3.2. Heterologous Expression Analysis of Mutant Pectate Lyase

To determine the enzymatic characteristics, prokaryotic expression was performed for mutant enzymes in *E. coli* BL21(DE3) under the same conditions as the wild type Pel419. SDS-PAGE analysis was conducted after the IPTG induction. Compared with the blank control group, the original enzyme Pel419 and five mutant enzymes all showed evident specific bands, and each recombinant mutagenic protein had an obvious band at 42 kDa, which was consistent with Pel419 (Figure S1).

### 3.3. Determination of the Optimal Catalytic Temperature

**Figure 3:**
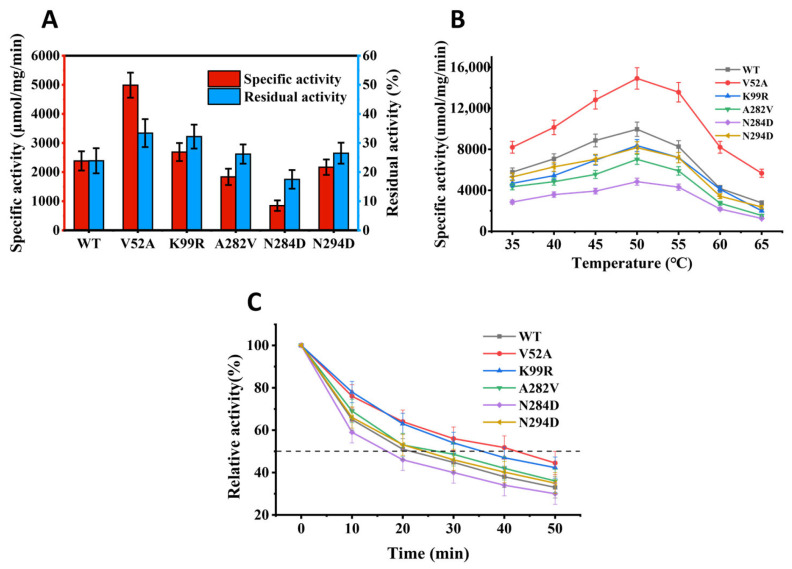
(**A**) Thermostability and specific activity of mutants. (**B**) The optimal reaction temperature of mutant enzyme compared to wild type Pel419. (**C**) Half-life of Pel419 and mutants at 50 °C. Each enzyme was assayed in 0.05 mol/L Gly-NaOH buffer (pH 9.0) using sodium polygalacturonic acid solution (5 mg/mL) as substrate enzymatic activity. The highest activity was taken as 100%. Values are the means ± SD of three replicates.

The optimal reaction temperatures of all enzymes to polygalacturonic acids were evaluated at different temperatures (35–65 °C). The influence of temperature on the enzymatic catalysis of polygalacturonic acids is shown in Figure 3B. All mutants presented the maximum activity at 50 °C, and their activity declined rapidly after the temperature exceeded 55 °C. The data showed that the optimal reaction temperature of enzymes was not influenced much by these mutants selected at the noncatalytic residues on the secondary structure.

### 3.4. Thermostability Test

After incubation at 50 °C for 1 h, the catalytic activity of all enzymes was reduced to different degrees. Following the treatment at 50 °C for 1 h, the residual activity of V52A was 33.4%, showing superior thermostability. In comparison to the wild type, the residual activity of K99R was also relatively improved, indicating that its thermostability was slightly enhanced. A282V and N294D presented lower residual activity than the wild type, but its thermostability was relatively elevated. N284D was relatively more sensitive to high temperature (Figure 3A) than the wild type. In addition, V52A effectively strengthened the thermostability of enzymes, as further proved by the half-life period (t_1/2_) of these pectate lyase at 50 °C (Figure 3C).

### 3.5. Ramie Degumming Effect

**Figure 4:**
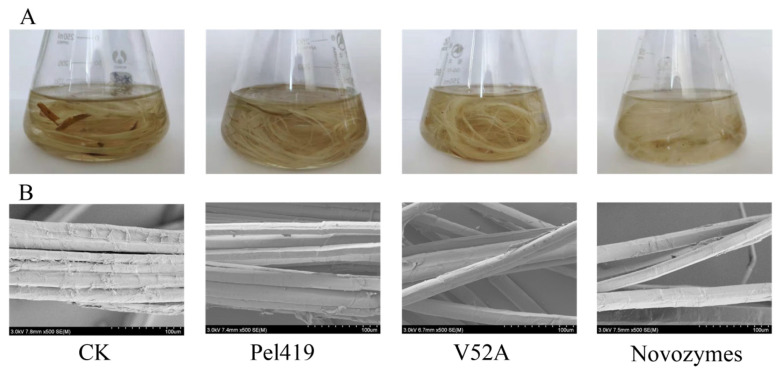
Surface morphology of the treated ramie. (**A**) Surface morphology of the untreated and treated ramie. (**B**) Scanning electron micrograph of the untreated and treated ramie.

The appearance morphology and fiber morphology of ramie were qualitatively analyzed after the pectate lyase treatment. The results in Figure 4A showed that the ramie fibers by the enzymatic treatment displayed favorable effects in flexibility, fiber dispersity and fiber whiteness. Compared with Pel419, the ramie fibers experiencing V52A treatment were of more eident dispersity, and their surface was smoother by the SEM observation (Figure 4B).

The ramie weight loss rate after different enzyme treatments were determined. As indicated by the results in Table 1, the ramie weight loss rate was the highest (19.22%) in the positive control treatment. The V52A treatment was 18.77%, being 5.88% higher than that in the treatment with the wild enzyme, and it is comparable with the industrially used enzyme.

## 4. Discussion

It is of great practical significance to apply pectate lyase to degumming bast fiber crops such as ramie. In fact, a series of pectate lyase from different microorganisms have been reported and commercialized, but more effective enzymatic characteristics are still lacking to satisfy large-scale industrial production [25,26,27,28]. To acquire enzymes with higher thermostability and specific activity, rational design based on the homology modeling structure has become an effective strategy for improving enzymatic performance [29]. Zhou et al. rationally designed *pel*N derived from *Paenibacillus* sp. 0602 to shift the optimal temperature of its enzymatic activity from 67.5 °C to 60 °C, and the half-life was increased by 15.9 min [30,31]. In this study, we constructed a series of proposed mutants by rational design. Finally, we obtained Pel419-V52A and Pel419-K99R with improved thermostability. Their optimum reaction temperature remained unchanged and their half-life increased significantly.

In fact, many studies have shown that certain mutational changes at residues displaying high B-factors and therefore high flexibility could result in thermostabilization [32]. For example, Bornscheuer et al. designed a clever sequence of mutagenesis experiments using B-FITTER for identifying residues with high B-factors, aiming for the thermostabilization of the esterase from *Pseudomonas fluorescens*. Following mutation, a variant was evolved showing a gain in thermostabilization [33]. In this study, based on multiple sequence alignment and homology modeling structure analysis (Figure 1 and Figure 2), the substrate binding pocket and the conservative sites in the structure were avoided, and a total of 5 mutation sites were determined combining the B-factor and mutation strategy of Pel419, and these recombinant enzymes were successfully expressed via *E coli* (Figure S1). Both wild type and mutant pectate lyases showed the maximum enzymatic activity at 50 °C (Figure 3B). 

**Figure 5:**
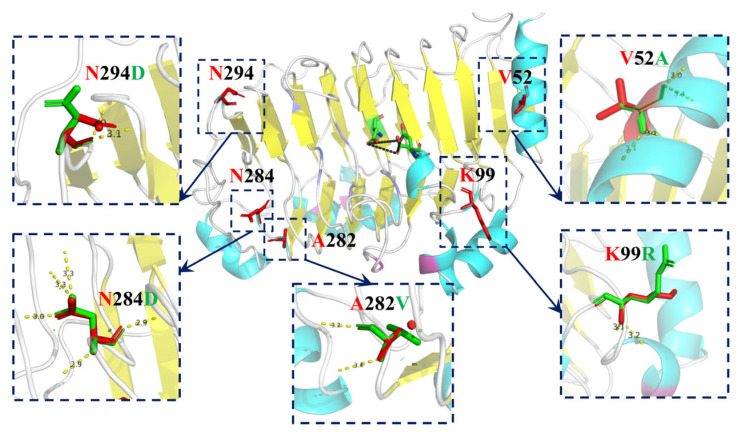
Molecular mechanism study of WT and mutants. Red and green sticks indicate pre- and post-mutation amino acids, respectively. Hydrogen bonds are represented as yellow dashed lines. All of the structural diagrams were drawn using PyMOL software.

Compared with the wild type, the thermostability of K99R was evidently enhanced, but the enzymatic activity was slightly impaired. Since Val is entropy penalized in the α-helix, the thermostability of V52A was improved efficiently (Figure 3A and Figure S4A). Homology modeling predictions for Pel419, then calcium and substrate, are grafted from 2EWE, where the K99 residue attached to the random coil was far away from the calcium ions and substrates, exceeding the distance of non-covalent bond interaction (Figure 5). However, when lysine is mutated to arginine, it is speculated that this will lead to a change in the dynamic correlation of the alpha helix adjacent to the mutated amino acid, which in turn indirectly affects the active center (Figure S3B) [34]. Meanwhile, K99 is located on the protein surface, and the hydrogen bonds between arginine residues and coordination water molecules increase after replacing, so as to strengthen the thermostability of proteins Figure 5 and Figure S4B) [35,36].

The thermostabilization trend of N294D and A282V was slightly higher than that of natural enzymes (Figure 3A). According to Figure 5 and Figure S4C, A282V mutated from Ala with a small molecular weight to Val with a relatively large molecular weight, which enhanced the rigidity of coil. N294D increases the number of hydrogen bonds (Figure S4E). N284D is relatively sensitive to high temperature and the enzyme activity is significantly reduced (Figure 3A) because Asp is an acidic amino acid, and the reaction under alkaline conditions will affect the enzyme activity. In addition, it is speculated that because N284D is located at the entrance of the substrate binding pocket, the group exposed to the protein surface changes from amino to carboxyl, which leads to the change of the charge on the protein surface. Meanwhile, because N284D is located at the exit of the substrate, the side chain carboxyl group of Asp residue binds to Ca^2+^ and hinders the release of substrates (Figure S4D).

Ramie degumming was implemented by selecting V52A with the best enzymatic activity and thermostability, and the ramie weight loss rate was 18.77%, which is comparable with the commercial enzymes, so V52A could be considered as a potential industrial enzyme applicable to the large-scale ramie degumming. Since the design and construction of new salt bridges is an effective method to improve the thermostability of proteins [37,38,39], in the follow-up study, the heat resistance of pectate lyase can be further transformed by combining the mutants V52A and K99R or introducing disulfide bonds and adding salt bridges, etc.

## 5. Conclusions

By comparing the enzymatic activity and heat resistance of five mutants, it was obtained that the enzymatic activity and thermostability of V52A were substantially improved. The thermostability of K99R was improved to a small extent, and its activity slightly declined. Despite the lower enzymatic activity than the wild type, A282V and N294D showed the similar thermostability to the wild type. The enzyme activity and thermal stability of N284D were decreased. The optimal reaction temperature of the five mutants was consistent with that of the wild type. Meanwhile, the ramie weight loss rate obtained by V52A at 50 °C is comparable with the commercial cotton-ramie processing pectate lyase, so V52A was regarded as a potential industrial enzyme applicable to the large-scale ramie degumming. Therefore, the enzymatic activity and heat resistance of Pel419 in alkaline environment were successfully transformed in this study, providing a potential material for developing ramie degumming enzyme preparations, and meanwhile, rendering a promising candidate method for the large-scale biotechnology applications aiming to improve the thermostability of pectate lyase.

## Figures and Tables

**Table 1:** Comparison on weight loss rate of ramie.

Enzymes	CK	Pel419	V52A	Novo
Weight loss rate (%)	4.77 ± 0.32	12.89 ± 0.28	18.77 ± 0.23	19.22 ± 0.21

Values are the means ± SD of three replicates.

## Data Availability

Publicly available datasets were analyzed in this study and can be found here: Protein (https://www.ncbi.nlm.nih.gov/protein/?term= accessed on 8 February 2022).

## Supplementary Materials

The following supporting information can be downloaded at: https://www.mdpi.com/article/10.3390/polym14142878/s1, Table S1: Highest B-factors of residues in homologous enzyme Pel419 as obtained by application of B-FITTER; Table S2: List of primers; Figure S1: SDS-PAGE analysis of crude solution of recombinant enzymes; Figure S2. Nucleic acid detection peak at mutation site; Figure S3. (A) Specific enzyme activity of wild enzyme and mutant enzyme. (B) Residual enzyme activity after holding at 50 °C for one hour. Values are the means ± SD of three replicates; Figure S4. Panels before and after mutation.

## References

[1] Nicole H., Guy C., Vladimir E.S. (2014). Bacterial pectate lyases, structural and functional diversity. Environ. Microbiol. Rep..

[2] Pan W., Yang S., Zhan Z., Zhang G. (2020). Origins and features of pectate lyases and their applications in industry. Appl. Microbiol. Biotechnol..

[3] Sonia A., Mandhan R.P., Sudha D.S., Rakesh K., Jitender S. (2008). Potential application of alkaline pectinase from *Bacillus subtilis* SS in pulp and paper industry. Appl. Biochem. Biotechnol..

[4] Nwobi A., Cybulska I., Tesfai W., Shatilla Y., Rodríguez J., Thomsen M.H. (2015). Simultaneous saccharification and fermentation of solid household waste following mild pretreatment using a mix of hydrolytic enzymes in combination with *Saccharomyces cerevisiae*. Appl. Microbiol. Biotechnol..

[5] Aggarwal R., Dutta T., Sheikh J. (2020). Extraction of pectinase from *Candida* isolated from textile mill effluent and its application in bio-scouring of cotton. Sustain. Chem. Pharm..

[6] Shukri N.A., Mohd Zin Z., MohdMaidin N., Hasmadi M., Zainol M.K. (2020). Ramification of pH in pectinase-assisted extraction on the antioxidant capacity of Arabica spent coffee ground (SCG) extract. J. Food Sci..

[7] Neelima M., Kavita S., Mukty S., Archana D., Brajesh P., Hyeji J., Seorin P., Srinath P., Sunghun C. (2021). Biotransformation of citrus waste-I: Production of biofuel and valuable compounds by fermentation. Processes.

[8] Zhang G., Li S., Xu Y., Wang J., Wang F., Xin Y., Shen Z., Zhang H., Ma M., Liu H. (2019). Production of alkaline pectinase: A case study investigating the use of tobacco stalk with the newly isolated strain *Bacillus tequilensis* CAS-MEI-2-33. BMC Biotechnol..

[9] Steven R.H., Robert D.S., Michael G., Margaret L., Frances J. (2003). Characterization and implications of Ca^2+^ binding to pectate lyase C. J. Biol. Chem..

[10] Marie-Line G., Miroslaw C. (2010). Structural and mechanistic classification of uronic acid-containing polysaccharide lyases. Glycobiology.

[11] Han C., Li W., Hua C., Sun F., Bi P., Wang Q. (2018). Enhancement of catalytic activity and thermostability of a thermostable cellobiohydrolase from *Chaetomium thermophilum* by site-directed mutagenesis. Int. J. Biol. Macromol..

[12] Cheng L., Duan S., Zheng K., Feng X., Yang Q., Liu Z.Y., Liu Z., Peng Y. (2019). An alkaline pectate lyase D from *Dickeya dadantii* DCE-01: Clone, expression, characterization, and potential application in ramie bio-degumming. Text. Res. J..

[13] López-Villamizar I., Cabezas A., Pinto R.M., Canales J., Ribeiro J.M., Rodrigues J.R., Costas M.J., Cameselle J.C. (2021). Molecular Dissection of Escherichia coli CpdB: Roles of the N Domain in Catalysis and Phosphate Inhibition, and of the C Domain in Substrate Specificity and Adenosine Inhibition. Int. J. Mol. Sci..

[14] Yury A.D., Alexander V.G., Aleksandra M.R., Dmitry O.O., Ivan N.Z., Veronika Y.M., Igor V.U., Arkady P.S. (2017). Site-directed mutagenesis of GH10 xylanase A from *Penicillium canescens* for determining factors affecting the enzyme thermostability. Int. J. Biol. Macromol..

[15] Chen X., Li W., Ji P., Zhao Y., Hua C., Han C. (2018). Engineering the conserved and noncatalytic residues of a thermostable β-1,4-endoglucanase to improve specific activity and thermostability. Sci. Rep..

[16] Wen Z., Zhang Z., Zhong L., Fan J., Li M., Ma Y., Zhou Y., Zhang W., Guo B., Chen B. (2021). Directed evolution of a plant glycosyltransferase for chemo- and regioselective glycosylation of pharmaceutically significant flavonoids. ACS Catal..

[17] Chen M., Song F., Qin Y., Han S., Rao Y., Liang S., Lin Y. (2022). Improving thermostability and catalytic activity of glycosyltransferase from panax ginseng by semi-rational design for rebaudioside D synthesis. Front. Bioeng. Biotechnol..

[18] Reetz M.T., Carballeira J.D., Vogel A. (2006). Iterative saturation mutagenesis on the basis of B factors as a strategy for increasing protein thermostability. Angew. Chem. Int. Ed. Engl..

[19] Laemmli U.K. (1970). Cleavage of structural proteins during the assembly of the head of bacteriophage T4. Nature.

[20] Bradford M.M. (1976). A rapid and sensitive method for the quantitation of microgram quantities of protein utilizing the principle of protein-dye binding. Anal. Biochem..

[21] Wang H.L., Li X.M., Ma Y.H., Song J.N. (2014). Characterization and high-level expression of a metagenome-derived alkaline pectate lyase in recombinant *Escherichia coli*. Process Biochem..

[22] Zhou Q., Ji P., Zhang J., Li X., Han C. (2017). Characterization of a novel thermostable GH45 endoglucanase from *Chaetomium thermophilum* and its biodegradation of pectin. J. Biosci. Bioeng..

[23] Liu Z., Duan S., Sun Q., Peng Y., Feng X., Zheng K., Hu Z., Zhang Y. (2012). A rapid process of ramie bio-degumming by *Pectobacterium* sp. CXJZU-120. Text. Res. J..

[24] Scavetta R.D., Herron S.R., Hotchkiss A.T., Kita N., Keen N.T., Benen J.A., Kester H.C., Visser J., Jurnak F. (1999). Structure of a plant cell wall fragment complexed to pectate lyase C. Plant Cell.

[25] Dinu D., Nechifor M.T., Stoian G., Costache M., Dinischiotu A. (2007). Enzymes with new biochemical properties in the pectinolytic complex produced by *Aspergillus niger* MIUG 16. J. Biotechnol..

[26] Wang H., Fu L., Zhang X. (2011). Comparison of expression, purification and characterization of a new pectate lyase from *Phytophthora capsici* using two different methods. BMC Biotechnol..

[27] Damak N., Abdeljalil S., Koubaa A., Trigui S., Ayadi M., Trigui-Lahiani H., Kallel E., Turki N., Djemal L., Belghith H. (2013). Cloning and heterologous expression of a thermostable pectate lyase from *Penicillium occitanis* in *Escherichia coli*. Int. J. Biol. Macromol..

[28] Atanasova L., Dubey M., Gruji M., Gudmundsson M., Lorenz C., Sandgren M., Kubicek C.P., Jensen D.F., Karlsson M. (2018). Evolution and functional characterization of pectate lyase PEL12, a member of a highly expanded *Clonostachys rosea* polysaccharide lyase 1 family. BMC Microbial..

[29] Xu Z., Cen Y., Zou S., Xue Y., Zheng Y. (2020). Recent advances in the improvement of enzyme thermostability by structure modification. Crit. Rev. Biotechnol..

[30] Zhou Z., Wang X. (2021). Rational design and structure-based engineering of alkaline pectate lyase from *Paenibacillus* sp. 0602 to improve thermostability. BMC Biotechnol..

[31] Zhou Z., Liu Y., Chang Z., Wang H., Leier A., Marquez-Lago T.T., Ma Y., Li J., Song J. (2017). Structure-based engineering of a pectate lyase with improved specific activity for ramie degumming. Appl. Microbiol. Biotechnol..

[32] Sun Z., Liu Q., Qu G., Feng Y., Reetz M.T. (2019). Utility of b-factors in protein science: Interpreting rigidity, flexibility, and internal motion and engineering thermostability. Chem. Rev..

[33] Jochens H., Aerts D., Bornscheuer U.T. (2010). Thermostabilization of an esterase by alignment-guided focussed directed evolution. Protein Eng. Des. Sel..

[34] Adrian B.H., Anderson J.L.R., Donald H., Arcus V.L., van der Kamp M.W., Mulholland A.J. (2021). Evolution of dynamical networks enhances catalysis in a designer enzyme. Nat. Chem..

[35] Blanes-Mira C., Blanesmira C., Ibañez C., Fernándezballester G., Planellscases R., Pérezpayá A.E. (2001). Thermal stabilization of the catalytic domain of *Botulinum neurotoxin* E by phosphorylation of a single tyrosine residue. Biochemistry.

[36] Nimpiboon P., Kaulpiboon J., Krusong K., Nakamura S., Kidokoro S., Pongsawasdi P. (2016). Mutagenesis for improvement of activity and thermostability of amylomaltase from *Corynebacterium glutamicum*. Int. J. Biol. Macromol..

[37] Donald J.E., Kulp D.W., DeGrado W.F. (2011). Salt bridges: Geometrically specific, designable interactions. Proteins.

[38] Ban X., Dhoble A.S., Li C., Zhang Y., Gu Z., Cheng L., Hong Y., Li Z. (2017). Potassium and sodium ions enhance the activity and thermostability of 1,4-alphaglucan branching enzyme from *Geobacillus thermoglucosidasius* in the presence of glycerol. Int. J. Biol. Macromol..

[39] Ban X., Wu J., Kaustubh B., Lahiri P., Dhoble A.S., Gu Z., Li C., Cheng L., Hong Y., Tong Y. (2020). Additional salt bridges improve the thermostability of 1,4-α-glucan branching enzyme. Food Chem..

