# Supplementary material for: Improvement on Thermostability of Pectate Lyase and Its Potential Application to Ramie Degumming
Source: Polymers (Basel). 2022 Jul 15;14(14):2878. doi: 10.3390/polym14142878 (PMC9318251; doi:10.3390/polym14142878)
Supplement: Supplementary file 1 [file polymers-14-02878-s001.zip › polymers-1772529-supplementary.pdf]

polymers MDPI

Supplementary Materials

Improvement on Thermostability of Pectate Lyase and Its Potential Application to Ramie Degumming

Huan Xu, Xiangyuan Feng, Qi Yang, Ke Zheng, Le Yi, Shengwen Duan * and Lifeng Cheng *

Institute of Bast Fiber Crops, Chinese Academy of Agricultural Science, Changsha 410205, China; xuhuan0310@yeah.net (H.X.); fengxiangyuan@caas.cn (X.F.); yangqi@caas.cn (Q.Y.); zhengke@caas.cn (K.Z.); yile0219@yeah.net (L.Y.).
* Correspondence: duanshengwen@caas.cn (S.D.); chenglifeng@caas.cn (L.C.); Tel.: +86-0731-88998516 (L.C.)

Table S1. Highest B-factors of residues in homologous enzyme Pel419 as obtained by application of B-FITTER.

residue	residue sequence no.	B-factor value	rank
Val	52	31	1
Lys	353	30	2
Asn	284	30	3
Gly	99	28	4
Asp	354	28	5
Lys	355	27	6
Asn	294	26	7
Asp	291	26	8
Asp	166	25	9
Ala	282	25	10

Table S2. List of primers.

Name and Site	Primer sequences
V52A	F: 5' GATGCAGGATATCGCGGATATCATCGCCGC 3'<br>R: 5' GCGGCGATGATATCCGCGATATCCTGCATC 3'
K99R	F: 5' CAATATCTGCGGCCAGTGGAGCCGTGACGCCCGCGGTGTG 3'<br>R: 5' CACACCGCGGGCGTCACGGCTCCACTGGCCGCAGATATTG 3'
A282V	F: 5' CTGGTTCGAAAACGTGGTGAACCCGGTGAC 3'<br>R: 5' GTCACCGGGTTCACCACGTTTTCGAACCAG 3'
N284D	F: 5' GTTCGAAAACGCGGTGGATCCGGTGACGTCCCGC 3'<br>R: 5' GCGGGACGTCACCGGATCCACCGCGTTTTCGAAC 3'
N294D	F: 5' GCTATGACGGTAAGGACTTCGGCACCTG 3'<br>R: 5' CAGGTGCCGAAGTCCTTACCGTCATAGC 3'

Mutated nucleotides are underlined and restriction sites are in bold.

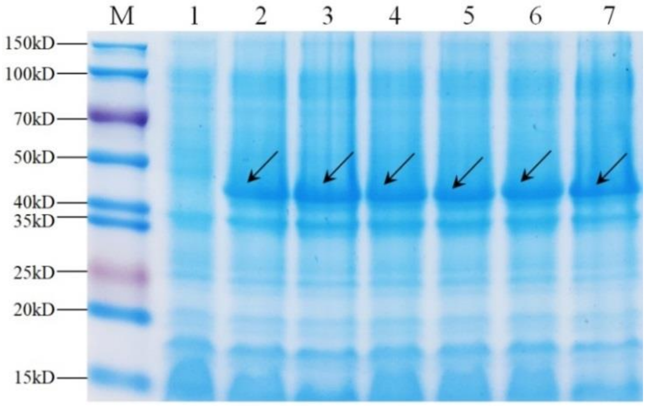

Figure S1. SDS-PAGE analysis of crude solution of recombinant enzymes. Lane M, molecular mass markers; lane; lane 1: empty plasmid; lane 2: wild type pel419; lane 3: V52A mutant; lane 4: K99R mutant; lane 5: A282V mutant; lane 6: N284D mutant; lane 7: N294D mutant.

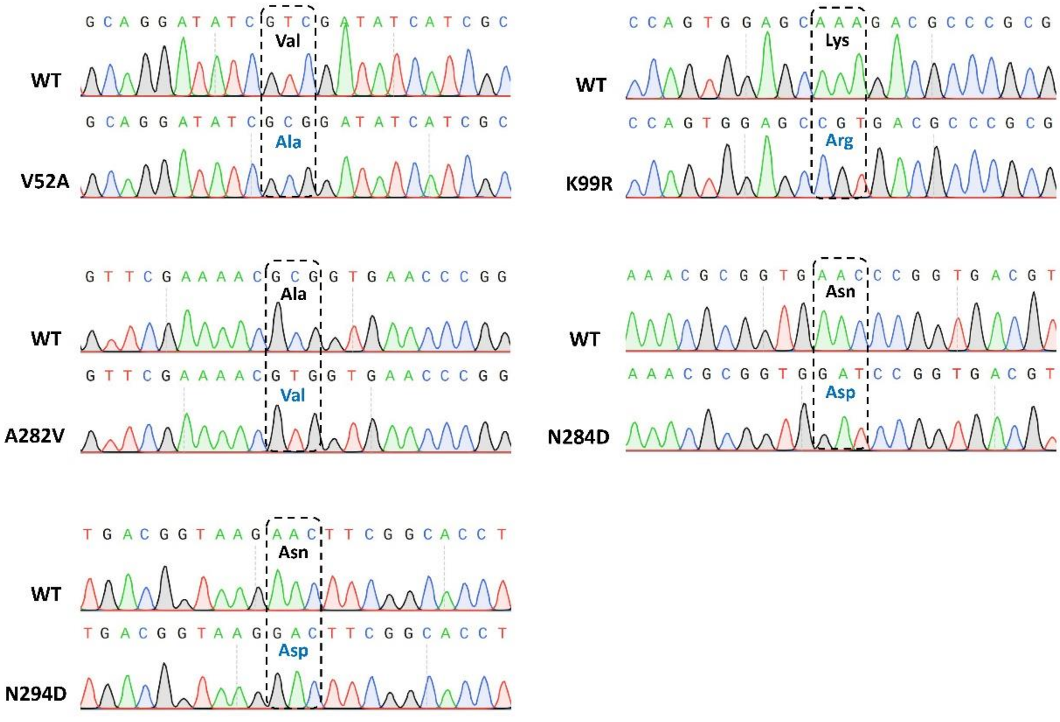

Figure S2. Nucleic acid detection peak at mutation site.

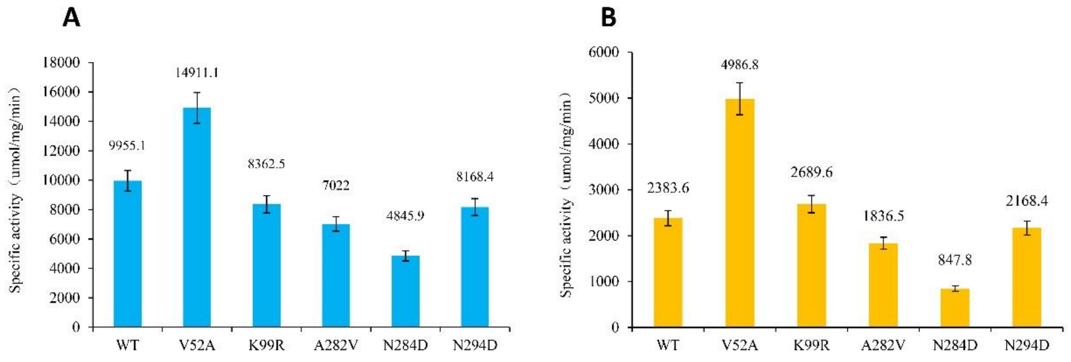

Figure S3. (A) Specific enzyme activity of wild enzyme and mutant enzyme. (B) Residual enzyme activity after holding at 50℃ for one hour. Values are the means ±SD of three replicates.

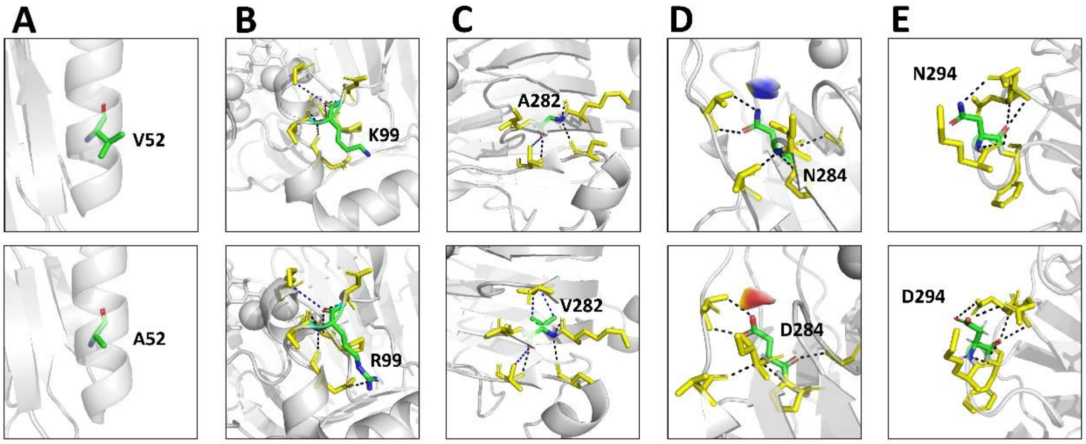

Figure S4. Panels before and after mutation. (A)Panel for V52residue. (B)Panel for K99 residue. (C)Panel for A282 residue. (D)Panel for N284 residue. (E)Panel for N294 residue.
